# mTORC2 Is Involved in the Induction of RSK Phosphorylation by Serum or Nutrient Starvation

**DOI:** 10.3390/cells9071567

**Published:** 2020-06-27

**Authors:** Po-Chien Chou, Swati Rajput, Xiaoyun Zhao, Chadni Patel, Danielle Albaciete, Won Jun Oh, Heineken Queen Daguplo, Nikhil Patel, Bing Su, Guy Werlen, Estela Jacinto

**Affiliations:** 1 Department of Biochemistry and Molecular Biology, Rutgers-Robert Wood Johnson Medical School, Piscataway, NJ 08854, USA; pcchou303@gmail.com (P.-C.C.); sr1208@scarletmail.rutgers.edu (S.R.); chadni.patel@gsbs.rutgers.edu (C.P.); dalbaciebio@gmail.com (D.A.); wjoh97@gmail.com (W.J.O.); hbd15@scarletmail.rutgers.edu (H.Q.D.); ndp85@scarletmail.rutgers.edu (N.P.); guy.werlen@rutgers.edu (G.W.); 2 Department of Immunology and Microbiology, Shanghai Jiao Tong University School of Medicine, Shanghai 200240, China; zhaoxiaoyunrf@163.com (X.Z.); bingsu@sjtu.edu.cn (B.S.)

**Keywords:** RSK, mTORC2, p90 ribosomal s6 kinase, nutrients, AGC kinases, MAPK/ERK, CCTβ, CCT/TRiC, chaperonin, starvation, metabolism

## Abstract

Cells adjust to nutrient fluctuations to restore metabolic homeostasis. The mechanistic target of rapamycin (mTOR) complex 2 responds to nutrient levels and growth signals to phosphorylate protein kinases belonging to the AGC (Protein Kinases A,G,C) family such as Akt and PKC. Phosphorylation of these AGC kinases at their conserved hydrophobic motif (HM) site by mTORC2 enhances their activation and mediates the functions of mTORC2 in cell growth and metabolism. Another AGC kinase family member that is known to undergo increased phosphorylation at the homologous HM site (Ser380) is the p90 ribosomal S6 kinase (RSK). Phosphorylation at Ser380 is facilitated by the activation of the mitogen-activated protein kinase/extracellular signal regulated kinase (MAPK/ERK) in response to growth factor stimulation. Here, we demonstrate that optimal phosphorylation of RSK at this site requires an intact mTORC2. We also found that RSK is robustly phosphorylated at Ser380 upon nutrient withdrawal or inhibition of glycolysis, conditions that increase mTORC2 activation. However, pharmacological inhibition of mTOR did not abolish RSK phosphorylation at Ser380, indicating that mTOR catalytic activity is not required for this phosphorylation. Since RSK and SIN1β colocalize at the membrane during serum restimulation and acute glutamine withdrawal, mTORC2 could act as a scaffold to enhance RSK HM site phosphorylation. Among the known RSK substrates, the CCTβ subunit of the chaperonin containing TCP-1 (CCT) complex had defective phosphorylation in the absence of mTORC2. Our findings indicate that the mTORC2-mediated phosphorylation of the RSK HM site could confer RSK substrate specificity and reveal that RSK responds to nutrient fluctuations.

## 1. Introduction

mTOR orchestrates metabolic processes in response to levels of nutrients in order to promote cell growth or survival [1,2,3]. It forms two distinct signaling complexes; mTOR complex 1 (mTORC1) and complex 2 (mTORC2). mTORC1 is composed of the evolutionarily conserved components mTOR, raptor, and mLST8 while mTORC2 contains mTOR, rictor, SIN1, and mLST8. In higher eukaryotes mTOR also associates with other proteins distinct from mTORC1 and mTORC2 [4,5]. mTOR is a serine/threonine protein kinase and its activity is modulated by its protein partners. The best-characterized substrate of mTORC2 is AKT which is a member of the AGC family of protein kinases [6]. Members of this family including AKT are phosphorylated at the kinase activation loop by PDK1 (phosphoinositide-dependent kinase 1) [7]. They are also phosphorylated at one or more sites at the two conserved motifs turn motif (TM) and hydrophobic motif (HM), which are adjacent to the kinase domain. There is accumulating evidence supporting that mTOR either as part of mTORC1 or mTORC2 phosphorylates directly or indirectly the TM and HM of AGC kinases [8,9,10,11,12,13,14,15]. mTORC2 phosphorylates the HM site (Ser473) of AKT in response to growth factors [10]. Recently we and others have also shown that this phosphorylation is enhanced upon nutrient withdrawal [16,17,18]. On the other hand, mTORC2 mediates phosphorylation of the TM of AKT as well as the HM/TM of PKCs constitutively [9,11,13,19,20]. These observations suggest that specificity of mTORC2 activity towards these targets is likely to be modulated compartmentally in response to levels of growth signals or intracellular metabolites. Indeed we found that the TM phosphorylation of AKT occurs during translation when nascent AKT is associated with translating ribosomes [19]. Identification of other downstream targets or effectors of mTORC2 should help unravel the precise mechanisms involved in mTORC2 signaling

**Figure 1:**
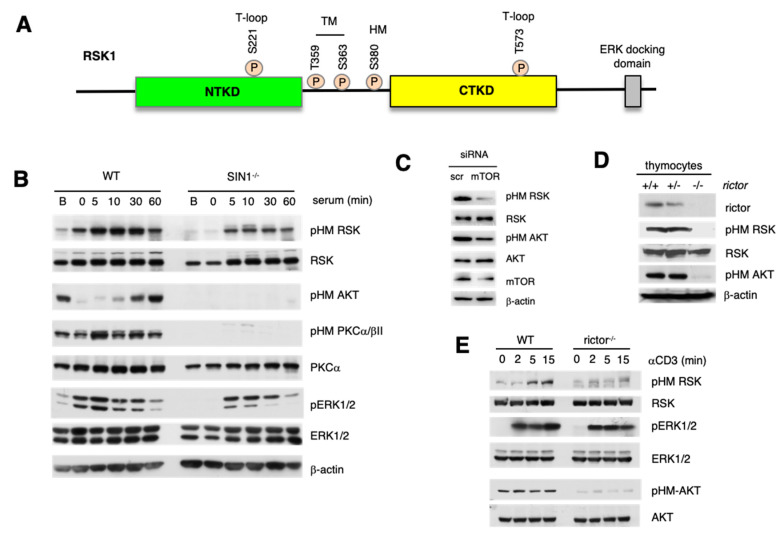
Phosphorylation of RSK at the hydrophobic motif site is diminished in mTORC2-disrupted cells. (**A**) RSK has two conserved catalytic domains, N-Terminal (NTKD) and C-Terminal (CTKD) kinase domains that are phosphorylated at each of their activation loops (T-Loop). These two domains flank a linker region that is conserved among AGC kinases containing the conserved turn (TM) and hydrophobic motifs (HM) that become phosphorylated at the indicated sites. (**B**) Wild type (WT) or SIN1^−/−^ MEFs were grown in complete DMEM (Basal; B) or grown then serum-starved overnight. Serum was re-added and cells were incubated for the indicated times (min) before harvest. Total lysates were prepared with CHAPS containing buffer and subjected to SDS-PAGE and immunoblotting using indicated antibodies. (**C**) HeLa cells were transfected with scramble control (scr) or siRNA targeting mTOR. Cells were lysed using RIPA and processed as in **B**. (**D**) Thymocytes with wild type (+/+), heterozygous (+/−) or homozygous (−/−) deletion of rictor were lysed and subjected to SDS-PAGE and immunoblotting. (**E**) Wild type (WT) or rictor-deficient thymocytes were non-stimulated (0) or stimulated with CD3ε antibody (10 μg/mL) for the indicated times (min).

The p90 ribosomal S6 kinase (RSK), another member of the AGC kinase family functions in translation, metabolism, cell adhesion/migration and becomes deregulated in diseases such as cancer [21,22,23,24,25,26]. RSK has different isoforms, RSK1–4, with distinct as well as overlapping functions. RSK1–4 consists of two kinase domains, the N-Terminal kinase domain (NTKD), which is homologous to the catalytic domain of AGC kinase family and another at the carboxyl terminus (CTKD), which is homologous to the calcium/calmodulin-dependent protein kinase (CaMK) family (Figure 1A). The CTKD and NTKD promote autophosphorylation and substrate phosphorylation, respectively [27,28]. The MAPK family member, ERK1/2, facilitates the activation of RSK. It docks at the C-terminal end and phosphorylates Thr573 of the CTKD activation loop [29]. ERK1/2 is also linked to phosphorylation of Ser363 at the TM, which is located at the linker region between the two kinase domains. This linker region harbors the conserved TM and HM of AGC kinases. Phosphorylation of Ser380 at the HM serves as a docking site for PDK1 that then phosphorylates Ser221 of the NTKD, resulting in full activation of RSK [30]. While HM site phosphorylation is strongly linked to ERK activation and could occur via autophosphorylation or ERK, the role of other kinases has not been excluded [26,31,32]. The combined removal of the ERK docking site and membrane targeting of RSK enhances HM phosphorylation and RSK activation, suggesting that the HM site is phosphorylated at the membrane [33]. In response to growth signals and mitogens, activated RSK phosphorylates a plethora of substrates [22,26]. Despite overlapping functions of RSK and other mTOR-regulated AGC kinases in a variety of cellular processes, the role of mTOR in RSK regulation remains unclear. In the present studies, we determined if mTORC2 could be involved in the regulation of RSK since the RSK NTKD harbors the homologous HM site that is targeted by mTORC2 in Akt, PKC, and SGK1 [9,11,12,13,14,34]. We unraveled that mTORC2 is required for optimal RSK HM site phosphorylation in the presence of growth signals but this function of mTORC2 does not require its catalytic activity. Importantly, we also found that RSK responds to nutrient starvation and this response is also mediated by mTORC2.

## 2. Materials and Methods

### 2.1. Plasmids and Antibodies

pKFLAG-CCTβ was obtained from Dr. John Blenis (Weill Cornell) [35] and HA-tagged avian RSK1 and Ser381 Ala mutant were obtained from Dr. Philippe Roux (IRIC, Univ. of Montreal) [29,33]. All other antibodies are listed in Table 1.

### 2.2. Cell Culture, Stimulation, Transfection and Harvest

HeLa, WT and SIN1^−/−^ MEFs were cultured in complete DMEM (Sigma D-6546)(St. Louis, MO, USA) [containing 10% FBS, 2 mM glutamine (Gibco 25030-164) (Gaithersburg, MD, USA), penicillin/streptomycin (Gibco 15140-122)]. After culturing for 24 h reaching approximately 70–80% confluency, cells were resuspended either in fresh complete media or starvation media (glucose starvation media-Corning 17-207-CV (Corning, NY, USA); glutamine starvation media-Corning 15-017-CV) as described previously [16]. 10% dialyzed FBS (Hyclone SH30079.03) (Marlborough, MA, USA), 25 mM glucose or 2 mM glutamine were supplemented in resuspension media as indicated. Cells were harvested with CHAPS lysis buffer (40 mM HEPES pH 7.5, 120 mM NaCl, 1 mM EDTA, 0.3% CHAPS) or RIPA lysis buffer (50 mM Tris-HCl pH 8.0, 100 mM NaCl, 5 mM EDTA, 0.2% SDS, 0.5% sodium deoxycholate, 1.0% Triton X-100) containing protease and phosphatase inhibitors. For transient transfections, plasmid constructs were transfected into MEFs (at about 60% confluency) using Lipofectamine Reagent (Invitrogen, Carlsbad, CA, USA) following the manufacturer’s protocol. After 24 h, cells were resuspended in DMEM lacking serum and incubated overnight. Cells were then resuspended in PBS for 30 min, then either harvested or restimulated with complete media containing 10% FBS followed by cell lysis. For siRNA transfections, cells were resuspended in Opti-MEM and incubated for 6–7 h. siRNA (mTOR siRNA Dharmacon L-003008-00; scramble Dharmacon D-001810-01-05) (Lafayette, CO, USA) was transfected using Oligofectamine (Invitrogen) following the manufacturer’s protocol. Twenty four hours after transfection, cells were harvested using RIPA lysis buffer.

### 2.3. Mice and Thymocyte Stimulation

The generation of mice with T cell-specific rictor deletion and thymocyte harvest were described previously [36]. For stimulation, thymocytes were incubated with 10 μg/mL CD3ε antibody and/or 10 ng/mL PMA. Handling and experimentation protocols have been reviewed and used in accordance with the Institutional Animal Care and Use Committee regulations of Rutgers University. 

### 2.4. Immunoblotting and Immunoprecipitations

Protein concentrations of cell lysates were determined by Bradford assay and samples (10–30 μg) were subjected to SDS-PAGE. Proteins were transferred onto Immobilon-PVDF (Millipore)(Burlington, MA, USA). Blots were incubated with primary antibodies overnight followed by washing in PBS-Tween. After incubation with secondary antibody for 1 h, blots were washed again. Images were visualized with a SuperSignal ECL detection kit (ThermoFisher, Waltham, MA, USA) and captured using a Amersham Imager 600 (GE)(Marlborough, MA, USA). For immunoprecipitations, lysates were Pre-Cleared by adding Protein G Sepharose beads (GE Healthcare, Marlborough, MA, USA), then allowed to tumble for 1 h at 4 °C. Supernatants were recovered then incubated with antibody overnight followed by an additional 1 h incubation with Protein G Sepharose beads at 4 °C. Beads containing immunoprecipitates were washed 3× with TBS (50 mM Tris-HCl pH 7.4, 150 mM NaCl). 

### 2.5. Immunofluorescence

WT MEFs were Co-Transfected with GFP-SIN1β and HA-RSK1-WT or HA-RSK1-SA mutant by PEI (Polyscience, 2,3966-1) (Niles, IL, USA). After 24 h, cells were changed into DMEM with 10%FBS. 24 h later, cells were resuspended in media with or without serum and incubated for 15 min. Cells were fixed using 1% PFA, blocked and stained for HA and DiI in PBS containing 1% BSA. Images were acquired on a Leica SP8 confocal Laser-Scanning microscope and processed using Bitplane Imaris 9.1.2 (Zurich, Switzerland).

## 3. Results

### 3.1. mTORC2 Is Required for Optimal Phosphorylation of RSK at the Hydrophobic Motif Site, Ser380

The phosphorylation of Ser380 at the HM of RSK is induced by growth factors and mediated by ERK [29]. Since this site is homologous to the HM site of Akt, we examined how the HM phosphorylation of RSK is affected in the absence of mTORC2. Using the mTORC2-disrupted cell line, SIN1^−/−^ murine embryonic fibroblasts (MEFs) [34], we examined RSK phosphorylation at Ser380 (based on human RSK1 numbering) (Figure 1A) (herein referred to as phospho-Hydrophobic Motif; pHM). Under basal conditions, pHM RSK was slightly lower in SIN1^−/−^ cells (Figure 1B). Upon resuspension in fresh media with serum, pHM RSK increased robustly from 5–30 min in WT while a blunted response occurred in SIN1^−/−^ MEFs. As expected, pHM of Akt and PKCα/βII was abolished in SIN1^−/−^ cells [9,34]. Since RSK phosphorylation at the HM site is dependent on activated ERK, which docks near the CTKD of RSK [37,38], we next examined ERK1/2 activation using the phosphorylation of ERK at Thr202/Tyr204 (pERK1/2) as the readout. ERK1/2 was activated upon serum restimulation in both WT and SIN1^−/−^ MEFs although this activation was slightly weaker in SIN1^−/−^ cells. Knockdown of mTOR in HeLa cells also diminished pHM RSK (Figure 1C). We also examined pHM RSK phosphorylation during the disruption of rictor, the other mTORC2 component. Using mice with specific deletion of rictor in thymocytes, we found that whereas pHM RSK was present in rictor^+/+^ and rictor^+/−^, it was abolished in the rictor^−/−^ thymocytes, similar to pHM AKT under basal conditions (Figure 1D). We then cultured thymocytes ex vivo and induced signaling downstream of the T cell receptor (TCR) by ligation of the CD3 subunit of the TCR using anti-CD3 antibody. pHM RSK was enhanced by 5 min of anti-CD3 stimulation in WT whereas it remained low in rictor^−/−^ thymocytes (Figure 1E). ERK1/2 was robustly stimulated in both WT and rictor^−/−^ thymocytes. Together, these findings indicate that mTORC2 modulates phosphorylation of the RSK HM phosphosite.

### 3.2. ERK Activation Is Essential but Not Sufficient for HM Site Phosphorylation of RSK

**Figure 2:**
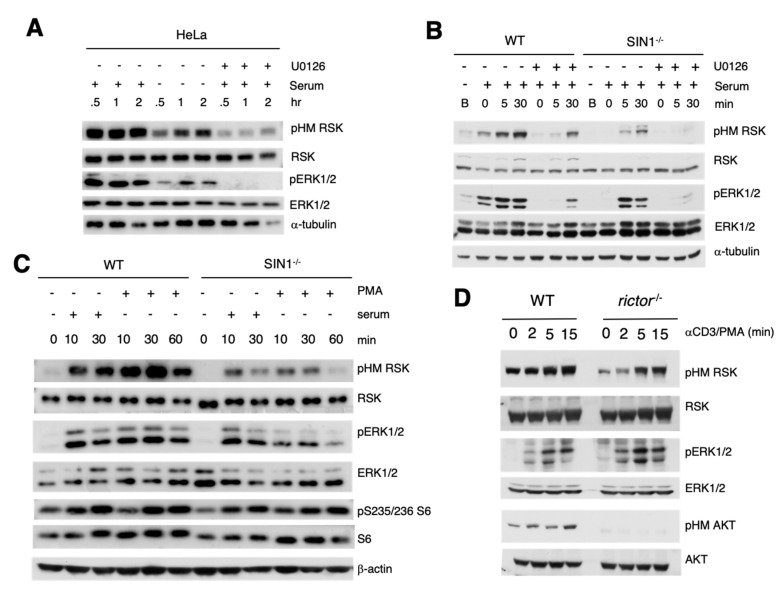
ERK activation is essential but not sufficient for full RSK HM site phosphorylation. (**A**) HeLa cells were resuspended in complete media without serum in the presence or absence of 15 μM U2016 for the indicated times. 1× FBS (serum) was added as indicated, at the last 0.5 h before harvest. Cells were harvested in CHAPS lysis buffer and protein extracts were subjected to SDS-PAGE and immunoblotting using indicated antibodies. (**B**) Wild type (WT) or SIN1^−/−^ MEFs were grown in complete DMEM (Basal; B) or grown then serum-starved overnight. Serum was re-added and cells were incubated for the indicated times (min). Cells were harvested and processed as in (**A**). (**C**) WT or SIN1^−/−^ MEFs were grown overnight in the absence of serum, followed by addition of serum or PMA for the indicated times. (**D**) WT or rictor^−/−^ thymocytes were non-stimulated (0) or stimulated with Anti-CD3 and 10 ng/mL PMA for the indicated times (min).

Since HM RSK phosphorylation was reduced but not abolished during stimulation of mTORC2-disrupted cells, we analyzed the contribution of ERK1/2 to this phosphorylation. We used the specific MEK inhibitor, U0126, which blocks ERK activation. This inhibitor diminished the serum-induced phosphorylation of the RSK HM site in HeLa, consistent with abrogation of ERK phosphorylation (Figure 2A). It also reduced pHM RSK in WT MEFs and abolished it in SIN1^−/−^ MEFs, correlating with abrogated ERK1/2 phosphorylation in the latter cells (Figure 2B). Thus, ERK1/2 activation, in addition to mTORC2-mediated phosphorylation is required for full RSK phosphorylation at the HM site. We next asked if enhancing activation of the ERK pathway by stimulation with the potent mitogen, phorbol myristate acetate (PMA), could rescue pHM RSK in SIN1-deficient cells. However, ERK activation and pHM RSK remained lower upon PMA stimulation in SIN1^−/−^ cells (Figure 2C). The phosphorylation of the RSK substrate, S6 at Ser235/236 in SIN1^−/−^ MEFs did not change significantly. 

We next examined whether pHM RSK in rictor^−/−^ thymocytes would be potentiated when we stimulate T cells with anti-CD3 and PMA. However, pHM RSK remained lower compared to WT (Figure 2D). On the other hand, ERK phosphorylation in the rictor^−/−^ thymocytes was comparable to WT. These findings indicate that although ERK activation is required, it is not sufficient and that mTORC2 is needed for optimal RSK HM site phosphorylation.

### 3.3. RSK HM Site Phosphorylation Is Increased during Nutrient Withdrawal via mTORC2

**Figure 3:**
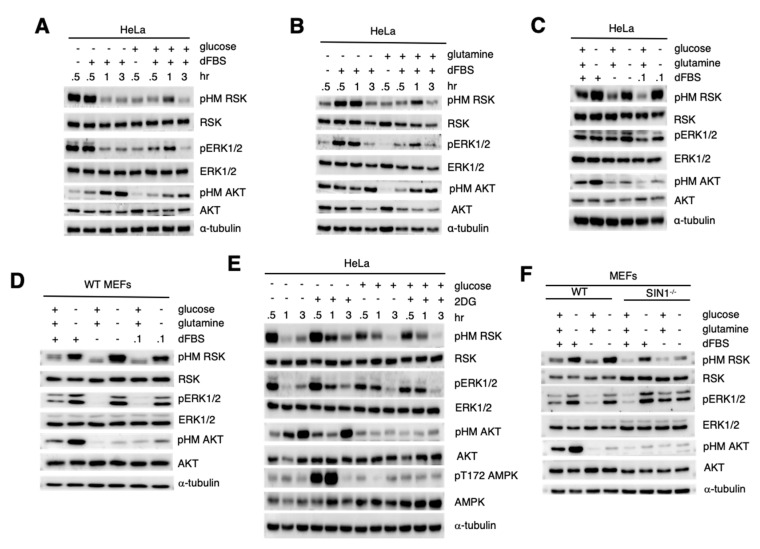
RSK HM site phosphorylation is increased during nutrient withdrawal. (**A**–**D**). Growing HeLa (**A**–**C**) or WT MEFs (**D**) were resuspended in media with or without 1X dialyzed FBS (dFBS) (or 1× dFBS as indicated in (**C**,**D**) and lacking (−) or containing (+) either glucose (**A**), glutamine (**B**) or both glucose and glutamine (**C**,**D**) at the indicated times. In (**C**,**D**), cells were harvested after 1 h. Cells were lysed with RIPA buffer and processed for SDS-PAGE and immunoblotting. (**E**) Growing cells were resuspended in media with dFBS in the absence or presence of glucose and/or 500 μM 2-Deoxyglucose (2DG) and incubated for the indicated times. (**F**) Growing WT or SIN1^−/−^ MEFs were resuspended in media lacking or containing glucose, glutamine and/or dFBS and incubated for 1 h before harvest. Cells were harvested and processed for SDS-PAGE and immunoblotting.

Since we have previously shown that mTORC2 is activated upon nutrient withdrawal [16], we then investigated how RSK-HM phosphorylation could be modulated by nutrients. We resuspended HeLa cells in media containing or lacking glucose, incubated them for 0.5–3 h with the addition of dialyzed serum for the last half hour before harvest. We found that pHM was more robust in the absence of glucose than when glucose was present, occurring transiently at 0.5 h (Figure 3A). The addition of serum did not further increase pHM when glucose was absent. Next, we incubated cells in the absence or presence of glutamine. Although RSK phosphorylation was low in the absence of both glutamine and serum, it was more robustly increased in the absence of glutamine upon re-addition of serum (Figure 3B). We next combined withdrawal of both glucose and glutamine in the presence or absence of serum. pHM was robustly increased when both nutrients were withdrawn in HeLa (Figure 3C) and WT MEFs (Figure 3D). Furthermore, withdrawal of all amino acids as well as glucose also led to a more robust HM phosphorylation in the absence or presence of serum (Figure S1). The increase in pHM also coincided with increased phosphorylation at the TM site as well as the NTKD and CTKD activation loop phosphosites (Figure S1). In all conditions tested (Figure 3A–D,Figure S1), the increase in RSK phosphorylation was accompanied by augmented ERK1/2 phosphorylation as well. 

Since glucose withdrawal robustly enhanced RSK phosphorylation, we then examined whether inhibition of glycolysis using 2-Deoxyglucose (2-DG) could also have the same effect. 2-DG did not affect RSK pHM in the presence of glucose but further increased RSK phosphorylation in the absence of glucose (Figure 3E). The strong effect of glucose starvation and glycolysis inhibition was also evident from the robust AMPK phosphorylation at 0.5–1 h. Together, these findings reveal that RSK HM site phosphorylation is robustly increased during nutrient withdrawal. 

We next examined whether the increase in HM RSK phosphorylation during nutrient starvation is also mediated by mTORC2. Compared to WT MEFs, pHM RSK was reduced in SIN1^−/−^ cells cultured for 1 h in the presence of glucose, glutamine and serum (Figure 3F). Upon withdrawal of both glucose and glutamine, pHM RSK was also lower in SIN1^−/−^ compared to the WT. However, when both glucose and glutamine were withdrawn in the absence of serum, pHM RSK was abrogated in SIN1^−/−^. Surprisingly ERK1/2 phosphorylation remained robust upon nutrient or serum withdrawal in SIN1^−/−^. Hence, pHM RSK is responsive to nutrient withdrawal and this response occurs via mTORC2.

### 3.4. Increased RSK HM Phosphorylation during Nutrient Withdrawal Is Uncoupled from S6 Phosphorylation

**Figure 4:**
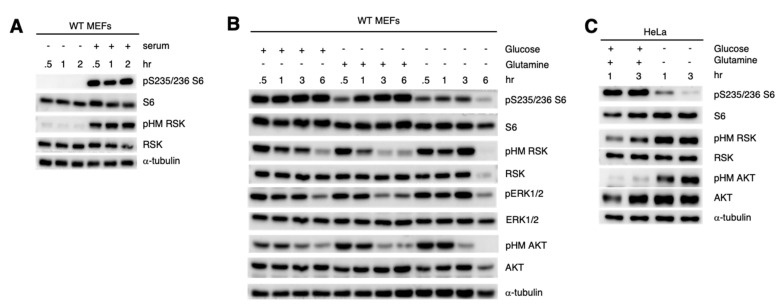
Phosphorylation of S6 is diminished despite increased RSK HM phosphorylation during nutrient withdrawal. (**A**) Growing WT MEFs were resuspended in complete media in the presence (+) or absence (−) of serum for the indicated times. Cells were harvested and processed for SDS-PAGE and immunoblotting. (**B**,**C**) Growing WT MEFs (**B**) or HeLa (**C**) were resuspended in media with dFBS and lacking either glucose, glutamine or both glucose and glutamine and incubated for the indicated times.

RSK phosphorylates the ribosomal protein S6 at Ser235/236 to modulate translation [39]. Indeed, upon serum restimulation of MEFs, we observed a robust phosphorylation of S6 that coincided with increased RSK HM phosphorylation (Figure 4A). When either glucose or glutamine were withdrawn for up to 6 h in WT MEFs, S6 phosphorylation remained robust (Figure 4B). However, when both glucose and glutamine were withdrawn from the culture media, S6 phosphorylation was greatly reduced whereas RSK HM phosphorylation was sustained for up to 3 h. We also examined the effect of combined glucose and glutamine withdrawal in HeLa and found that S6 phosphorylation was abolished by 3 h starvation whereas pHM RSK phosphorylation remained high at this point (Figure 4C). Furthermore, whereas S6 phosphorylation was similarly induced upon serum restimulation of serum-starved vs. serum/nutrient-starved cells, pHM RSK was more robust in the latter condition (Figure S1). Thus, the increased RSK HM phosphorylation that occurs during nutrient starvation is not linked to the phosphorylation of the RSK substrate, S6.

### 3.5. The Catalytic Activity of mTOR Is Not Required for RSK HM Site Phosphorylation

**Figure 5:**
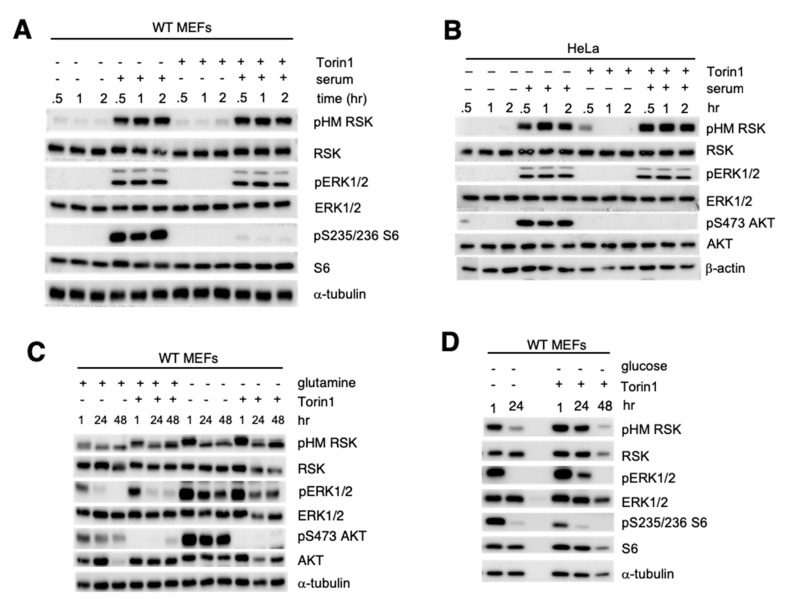
The catalytic activity of mTOR is not required for RSK HM site phosphorylation. (**A**,**B**) Growing WT MEFs (**A**) or HeLa cells (**B**) were supplemented with either 1 μM Torin1, 1× FBS (serum) or both and incubated for the indicated times. (**C**,**D**) Growing WT MEFs were resuspended in media with dFBS, lacking (−) or containing) glutamine (**C**) or glucose (**D**). Torin1 (1 μM) or vehicle (−) was added during resuspension and incubated at the indicated times.

To further define how RSK HM phosphorylation is modulated by mTORC2, we used the mTOR ATP-Competitive inhibitor, Torin1, which blocks all mTOR complex activity. Treatment of WT MEFs with Torin1 in the presence of serum did not diminish RSK HM phosphorylation (Figure 5A). There was also no effect of Torin1 on serum-induced pHM RSK nor ERK phosphorylation in HeLa cells (Figure 5B). On the other hand, Torin1 inhibited the phosphorylation of S6 and Akt (Figure 5A,B). As expected, rapamycin, which allosterically inhibits mTORC1, also failed to block the serum-induced HM RSK phosphorylation (Figure S2) [40]. Next, we examined if mTOR inhibition would affect nutrient starvation-induced pHM RSK. In WT MEFs, Torin1 did not abolish the phosphorylation induced by glutamine withdrawal (Figure 5C). Similarly, pHM RSK was also higher and even sustained up to 24 h under glucose starvation in Torin1-Treated WT MEFs (Figure 5D). Hence, the catalytic activity of mTOR is not essential in promoting RSK HM phosphorylation and that inhibition of mTOR kinase activity could instead sustain phosphorylation at this site during prolonged starvation.

### 3.6. RSK and SIN1 Colocalize at the Plasma Membrane

**Figure 6:**
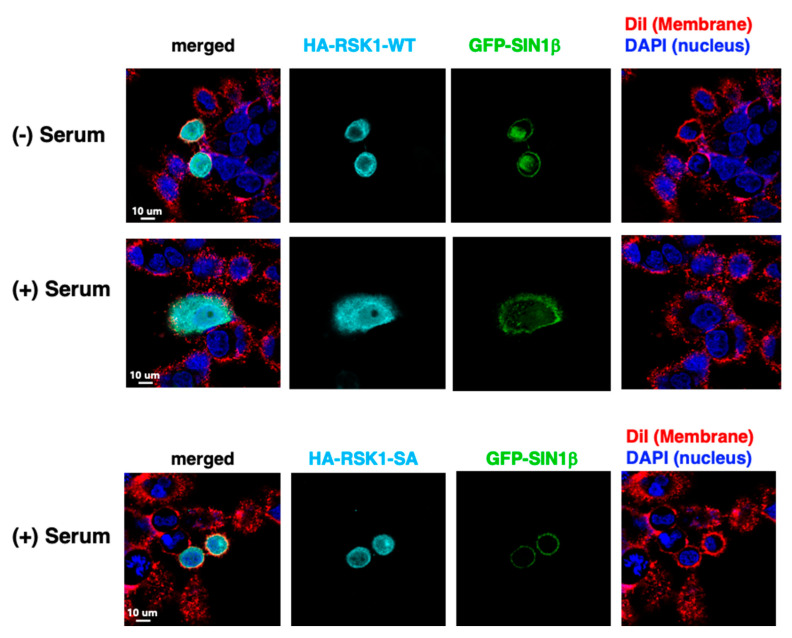
RSK and SIN1 colocalize at the plasma membrane. WT MEFs were co-transfected with GFP-SIN1β and avian HA-RSK-WT or HA-RSK-SA mutant (Ser381 Ala). After 48 h, cells were restimulated with serum for 15 min. Staining and microscopy were performed to detect GFP, HA as well as DiI (membrane) and DAPI (nucleus).

To further examine the role of the mTORC2-mediated RSK phosphorylation, we co-expressed GFP-SIN1β with either the wild type avian HA-RSK1 (HA-RSK1-WT) or the phospho-deficient mutant HA-RSK1-Ser381Ala (HA-RSK1-SA) that was devoid of HM phosphorylation even after serum restimulation (Figure S3). In the absence of serum, cells were more rounded in morphology (Figure 6). GFP-SIN1β localized to the membrane and the nucleus whereas HA-RSK1-WT localized to the plasma membrane and perinuclear area. Upon serum stimulation, there was more cell-spreading in HA-RSK1-WT-expressing cells. Whereas GFP-SIN1β predominantly localized on the plasma membrane in the presence of serum, HA-RSK1-WT was more diffused and present throughout the cell. On the other hand, when the mutant HA-RSK1-SA was expressed, cells remained round with less cell-spreading even in the presence of serum. Although GFP-SIN1β still localized to the membrane, HA-RSK1-SA localization was less diffused and predominated on the membrane. Next, we withdrew glutamine from the media. HA-RSK and GFP-SIN1 colocalized at the plasma membrane at 30 min glutamine withdrawal (Figure S4). HA-RSK-WT localization was more diffused in the presence of glutamine or by 6 h starvation. On the other hand, HA-RSK-SA mutant had diffused localization at all time points whereas SIN1 remained present at the membrane. These findings indicate that SIN1 and RSK colocalize at the membrane during serum restimulation and acute glutamine withdrawal. We then examined if RSK and SIN1 could interact. Immunoprecipitated Myc-SIN1 had increased association with HA-RSK1 upon withdrawal of both glucose and glutamine (Figure S5). Together, these findings suggest that mTORC2 could act as a scaffold to allow RSK phosphorylation in the membrane in response to serum or nutrient starvation.

### 3.7. The RSK Substrate, CCTβ, Has Defective Phosphorylation in the Absence of mTORC2

**Figure 7:**
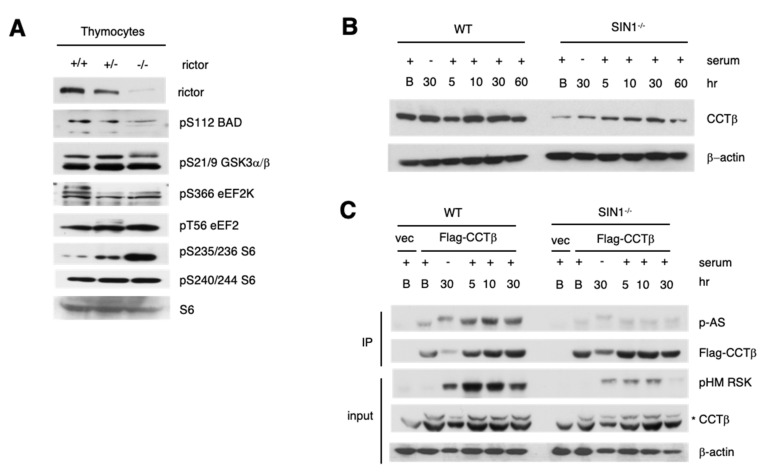
The RSK substrate CCTβ has defective phosphorylation and expression in the absence of mTORC2. (**A**) Thymocyte lysates from wild type (rictor^+/+^), rictor^+/−^, or rictor^−/−^ littermates were resolved by SDS-PAGE and analyzed for phosphorylation of known RSK substrates by immunoblotting. (**B**) Wild type (WT) or SIN1^−/−^ MEFs were grown in complete DMEM (Basal; B) or grown then serum-starved overnight. Cells were then left starved (–) or serum was re-added and cells were incubated for the indicated times. (**C**) WT or SIN1^−/−^ MEFs were transfected with Flag-CCTβ plasmid or vector control (vec). Cells were starved and re-stimulated with serum as in **B**. Lysates were subjected to immunoprecipitation using Flag antibody. Immunoprecipitates were fractionated and blotted for CCTβ phosphorylation (using the Phospho-Akt consensus motif substrate antibody (P-AS)) or Flag. Total extracts (input) were also fractionated by SDS-PAGE and immunoblotted for pHM RSK or CCTβ. (*) indicates exogenous Flag-CCTβ, lower band is endogenous CCTβ.

RSK phosphorylates various substrates in response to growth stimuli. To address how mTORC2 could affect phosphorylation of known RSK targets, we used the mTORC2-disrupted cells and compared phosphorylation of some of these targets. In the Rictor-deficient murine thymocytes, we found that only the phosphorylation of the apoptosis regulator, BAD, was diminished as compared to wild type cells (Figure 7A). Other known RSK targets including pS21/9 GSK3α/β, pS366 eEF2K and pT56 eEF2 [22] did not have discernible changes in phosphorylation. Interestingly, the phosphorylation of S6 at the RSK-targeted site, Ser235/236, was upregulated in the rictor^−/−^ thymocytes whereas the S6K1/mTORC1-mediated phosphorylation, Ser240/244 S6, was not altered. Another target of RSK that undergoes phosphorylation at the consensus Akt phosphorylation motif (RXRXXpS/pT) is CCTβ, a subunit of the chaperonin T-Complex protein-1 ring complex (TRiC/CCT) [35]. We first analyzed the expression of CCTβ in SIN1^−/−^ MEFs and found that its total protein levels were diminished in these cells (Figure 7B). To facilitate analysis and comparison of CCTβ phosphorylation in WT vs. SIN1^−/−^ MEFs, we overexpressed Flag-CCTβ, then analyzed the phosphorylation of immunoprecipitated CCTβ. Using the Phospho-Akt substrate antibody (P-AS), which detects phosphorylated epitope corresponding to the consensus RXRXXpS/T, we found that whereas CCTβ from the WT increased its phosphorylation upon serum addition, immunoprecipitated CCTβ from the SIN1^−/−^ MEFs had no discernible phosphorylation even up to 30 min of serum stimulation (Figure 7C). Hence, the phosphorylation of CCTβ at the RSK-targeted site is dependent on mTORC2. 

## 4. Discussion

**Figure 8:**
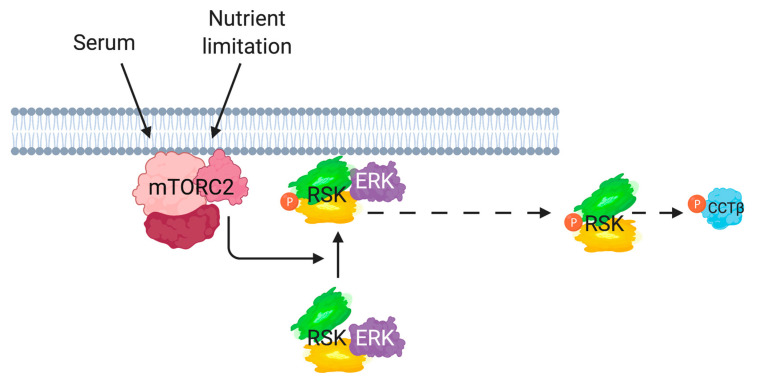
Model of mTORC2 regulation of RSK. In response to serum/growth factor stimulation or upon nutrient withdrawal, RSK phosphorylation (shown in orange) at the hydrophobic motif site (Ser380), located at the linker region between the NTKD (green) and CTKD (yellow), requires an intact mTORC2 but not mTOR catalytic activity. SIN1 associates with and colocalizes with RSK at the plasma membrane. mTORC2 could serve as a scaffold to potentiate the CTKD-mediated RSK HM phosphorylation. The CTKD is activated by ERK. mTORC2 is also required for the phosphorylation of the RSK substrate CCTβ.

mTORC2 regulates cell growth and metabolism in response to levels of growth factors and nutrients. Among its known targets are protein kinases that are members of the AGC kinase family including AKT, PKC, and SGK1 [8,41]. Here, we found that mTORC2 could also mediate the phosphorylation of another AGC kinase, RSK, at the conserved HM site Ser380 (Figure 8). However, unlike its role in modulating other AGC kinases, the catalytic activity of mTORC2 is not required to enhance the RSK HM site phosphorylation and may instead act as a scaffold to allow RSK phosphorylation at the membrane. Among some of the known substrates of RSK that we examined, only the Pro-Apoptotic protein, BAD, and the chaperonin complex subunit, CCTβ, had diminished phosphorylation in the absence of mTORC2. Similar to the regulation of AKT by mTORC2, RSK phosphorylation is also increased during nutrient withdrawal, suggesting a role for RSK in metabolic reprogramming during nutrient-limiting conditions. 

First, we found that mTORC2 is required for optimal phosphorylation of RSK at Ser380, which is located at the HM. This site is flanked by two catalytic domains, the NTKD and CTKD, which are phosphorylated at the activation loop (T-Loop) by PDK1 and ERK, respectively [22,26]. These two domains are linked by a region that harbors the TM and HM that are common to many AGC kinases. Phosphorylation of the RSK HM site is believed to be via autophosphorylation, mediated by the CTKD [33]. Stimulation of cells with growth factors or mitogens activates ERK and triggers its phosphorylation of the RSK CTKD activation loop. Previous studies have revealed the importance of ERK activation to pHM but whether another kinase is involved in enhancing this phosphorylation has not been excluded. The phosphorylation of the CTKD by ERK may facilitate membrane translocation of RSK, where it can be further activated in this compartment [33]. Loss of the ERK docking site located at the C-terminus abolishes RSK activity but a C-terminally truncated form that is targeted to the membrane results in HM phosphorylation and RSK activation [33], suggesting a critical role of membrane targeting for RSK regulation. Our studies here reveal that mTORC2 could mediate the regulation of RSK at the membrane. Although the catalytic activity of mTORC2 is not required for the HM site phosphorylation (Figure 5), mTORC2 could instead recruit or anchor RSK at the membrane to allow phosphorylation at this site. Phosphorylated HM serves as a docking site for PDK1, which then phosphorylates the T-Loop of the NTKD, leading to full RSK activation [42,43]. Upon activation at the membrane, RSK redistributes to other cellular compartments including the nucleus where it targets its many substrates [33]. mTORC2 has been found to associate with membrane compartments including the plasma membrane, lysosomes, nucleus, ER, and at the mitochondria-associated ER membrane [44,45,46,47]. In particular, SIN1 contains a pleckstrin homology (PH) domain that binds phosphatidylinositol 3,4,5-trisphosphate (PIP3) and as we show here localizes predominantly at the plasma membrane (Figure 6) [48]. The mTOR complexes, either directly or indirectly, mediate the phosphorylation of a number of the AGC kinases in a membrane compartment. In this regard, the subcellular localization of mTORC2 has been shown to impart specificity towards AGC kinase phosphorylation [49]. Here, we have shown that SIN1 and HM-phosphorylated RSK colocalize at the membrane during serum restimulation and acute glutamine withdrawal (Figure 6 and Figure S4). We also found increased association of RSK1 with SIN1 during nutrient starvation (Figure S5). Whether RSK associates with SIN1 and other mTORC2 components at other membrane compartments remains to be examined further. It is also possible that mTORC2 could mediate RSK phosphorylation via modulation of ERK signaling [50,51]. SIN1 physically associates with Ras and there is accumulating evidence on the interaction of mTORC2 with Ras/MAPK signaling [52,53,54,55]. Further studies are needed to elucidate how these two pathways cooperate to modulate RSK and other downstream targets to promote cell growth and survival. Our findings here expand the mTORC2 effectors among the AGC kinase family that become phosphorylated in a membrane compartment.

Second, we report here that RSK HM phosphorylation is robustly increased by nutrient withdrawal, a condition that also activates mTORC2 (Figure 3). We have previously demonstrated that the increase in mTORC2 activation during prolonged starvation enhances or maintains flux through critical metabolic pathways [16,56]. mTOR is a key signaling protein that responds to nutrient fluctuations. Whereas mTORC1 activation is promoted by the presence of nutrients, the mode of mTORC2 activation is context dependent. mTORC2 has basal activity that allows constitutive phosphorylation of the TM site of AKT and PKC [9,11,19]. On the other hand, its activation is enhanced by either restimulation of starved cells with growth factors or by withdrawal of nutrients. As we have shown here, RSK HM site phosphorylation also follows this mode of regulation. It is noteworthy that a previous study reported that RSK and ERK phosphorylation was enhanced upon amino acid restimulation of cells that have been starved of both serum and amino acids for prolonged periods [57]. We have also observed a more robust increase in phosphorylation of RSK at the HM, TM and activation loop sites upon restimulation of cells starved of both serum and amino acids as compared to serum-starved alone (Figure S1). However, what we found here was that the withdrawal of nutrients (either glucose, glutamine or both) could transiently increase RSK HM phosphorylation (Figure 3 and Figure 4B). Declining glucose levels were also shown to enhance RSK phosphorylation as well as ERK activation [18]. The findings by Casas-Terradellas et al. are not necessarily contradictory to our results and those from Shin et al. [18,57]. It is possible that similar to AKT which responds to either the increase or decrease of growth signals [16,17,18,34,58], RSK is also modulated by nutrient fluctuations and suggest that RSK is involved in remodeling metabolic processes. RSK has been previously shown to regulate PFKFB2 (6-phosphofructo-2-kinase/fructose-2,6-bisphosphatase 2) to maintain flux through glycolysis in melanoma cells [21]. Highly proliferating cells such as cancer cells upregulate signaling pathways that control nutrient availability and flux through metabolic pathways in order to meet the increased demand for macromolecules [59]. Hence, the activation of RSK during nutrient withdrawal is likely relevant during metabolic reprogramming of cancer cells. Future studies should address additional targets of RSK in nutrient acquisition and metabolism.

Third, we uncover that the RSK substrate CCTβ is also modulated by mTORC2. CCTβ, which is part of the multi-protein chaperonin complex (TRiC/CCT) is involved in folding of nascent polypeptides such as those involved in the cytoskeleton [60,61]. Although RSK phosphorylates various substrates, including transcription factors, translational regulators, enzymes, and structural proteins [26], we found that among the RSK substrates that we examined, only CCTβ displayed a profound defect in mTORC2-disrupted cells. Both the phosphorylation of CCTβ and its expression levels were diminished in the absence of mTORC2. CCTβ Ser260 was previously identified as the RSK-targeted site [35]. However, other AGC kinases including S6K1 and Akt could also phosphorylate this site depending on stimulatory conditions. Since Akt activation is also defective in SIN1^−/−^ MEFs, whereas S6K1 phosphorylation is not [34], it is possible that the suboptimal Akt activation contributes to the aberrant CCTβ phosphorylation. How phosphorylation of CCTβ by these mTORC-regulated kinases affects CCT function distinctly remains to be further investigated. Recently, CCT was also shown to mediate the assembly of the mTOR complexes [62]. Hence, it is also possible that there is feedback regulation between the mTORCs and CCT.

RSK and mTOR are important drug targets for diseases including cancer, cardiovascular and neurological disorders [22,63]. Our studies unravel that mTORC2 modulates RSK. Future studies should unravel the precise mechanisms that promote RSK activation by mTORC2 since they could have therapeutic value for targeting diseases with deregulated RSK signaling.

## Figures and Tables

**Table 1:** List of Antibodies used.

Target	Catalog No.	Source
pS380 RSK	AF889	R&D Systems (Minneapolis, MN, USA)
pS380 RSK (pHM)	12032	Cell Signaling Tech. (Danvers, MA, USA)
RSK	9347, 2765	Cell Signaling Tech.
pT359/S363 RSK (pTM)	9344	Cell Signaling Tech.
pT573 RSK	9346	Cell Signaling Tech.
pS221 RSK	AF892	R&D Systems
pThr202/Tyr204 ERK	4370, 2331	Cell Signaling Tech.
ERK	Sc-13003	Sta. Cruz Biotech. (Dallas, TX, USA)
pS235/236 S6	4856	Cell Signaling Tech.
S6	2317	Cell Signaling Tech.
pS473 Akt (pHM)	4060	Cell signaling Tech
Akt	9272	Cell Signaling Tech.
pT638/641 PKCα/βII (pHM)	9375	Cell Signaling Tech.
rictor	9476	Cell Signaling Tech.
β-actin	sc-47778	Sta. Cruz Biotech.
α-tubulin	sc-53029	Sta. Cruz Biotech.
pT172 AMPK	2535	Cell Signaling Tech.
AMPK	5831	Cell Signaling Tech.
pS112 BAD	9291	Cell Signaling Tech.
pS21/9 GSK3α/β	9331	Cell Signaling Tech.
pS366 eEF2K	2331	Cell Signaling Tech.
pT56 eEF2	2331	Cell Signaling Tech.
pS240/244 S6	2215	Cell Signaling Tech.
p-Akt substrate (pAS)	9614	Cell Signaling Tech.
CCTb	sc-13874	Sta. Cruz Biotech.
Flag	F7425	Sigma (St. Louis, MO, USA)
HA	3724	Cell Signaling Tech.
DiI	D282	Invitrogen (Carlsbad, CA, USA)
CD3ε	100302	Biolegend (San Diego, CA, USA)

## Supplementary Materials

The following are available online at https://www.mdpi.com/2073-4409/9/7/1567/s1, Figure S1: There is increased RSK HM site phosphorylation upon removal of amino acids, Figure S2: RSK HM site phosphorylation is not inhibited by rapamycin, Figure S3: RSK-Ser381Ala mutant is not phosphorylated during serum restimulation, Figure S4: RSK-WT colocalizes with SIN1 at the membrane during early nutrient withdrawal, Figure S5: There is increased association of RSK and SIN1 during glucose/glutamine withdrawal.
