# Supplementary material for: mTORC2 Is Involved in the Induction of RSK Phosphorylation by Serum or Nutrient Starvation
Source: Cells. 2020 Jun 27;9(7):1567. doi: 10.3390/cells9071567 (PMC7408474; doi:10.3390/cells9071567)
Supplement: Supplementary file 1 [file cells-09-01567-s001.pdf]

Chou et al.
Supplement Figures

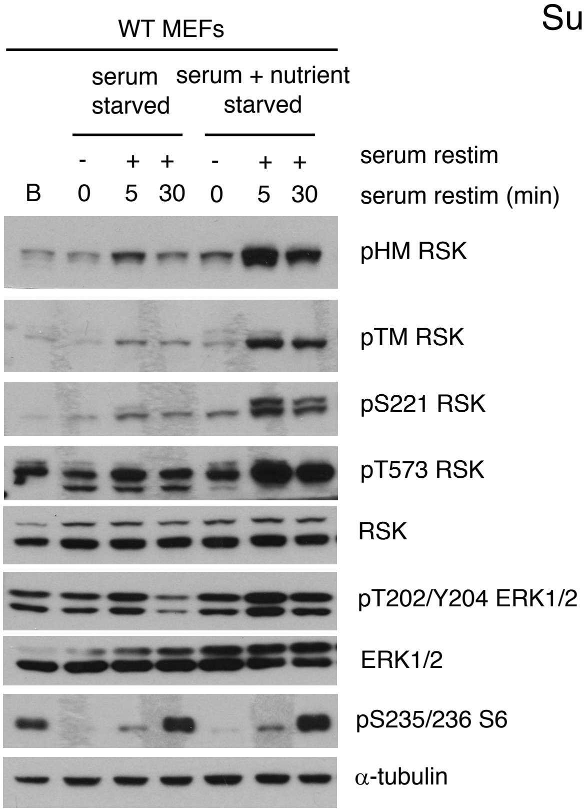

Figure S1: There is increased RSK HM site phosphorylation upon removal of amino acids. Growing wild type MEFs were either resuspended in fresh complete media (Basal; B) or in media lacking serum and incubated overnight. The following day, serum-starved group were either kept under the same media (unchanged) or resuspended in PBS for 1 hr (serum, amino acid, nutrient starved). After 1 hr, cells were either harvested (0 min) or restimulated with 10% serum for the indicated time, followed by harvesting, SDS-PAGE and immunoblotting.

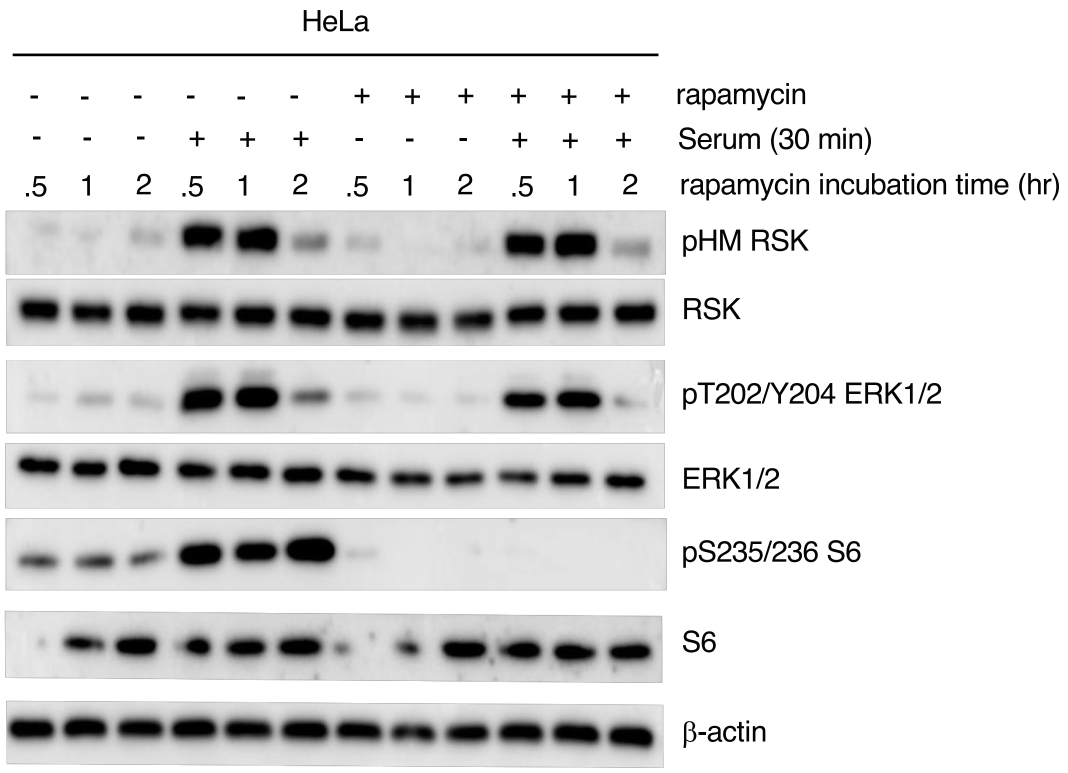

Figure S2: RSK HM site phosphorylation is not inhibited by rapamycin.
Growing wild type HeLa cells were resuspended in media lacking serum. Rapamycin (100 nM) or vehicle (-) was added to culture and incubated for the indicated times. Serum was added at the last half hour before harvest.

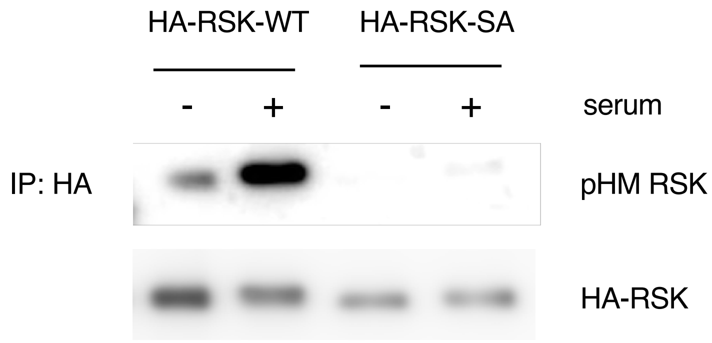

Figure S3: RSK-Ser381Ala mutant is not phosphorylated during serum stimulation.
Growing wild type MEFs were transfected with either HA-RSK1 WT or HA-RSK-Ser381Ala mutant constructs. The following day, cells were resuspended in media lacking or containing serum. After 15 min incubation, cells were harvested and total extracts were subjected to immunoprecipitation using HA antibody. Immunoprecipitated proteins and total proteins were fractionated by SDS-PAGE followed by immunoblotting.

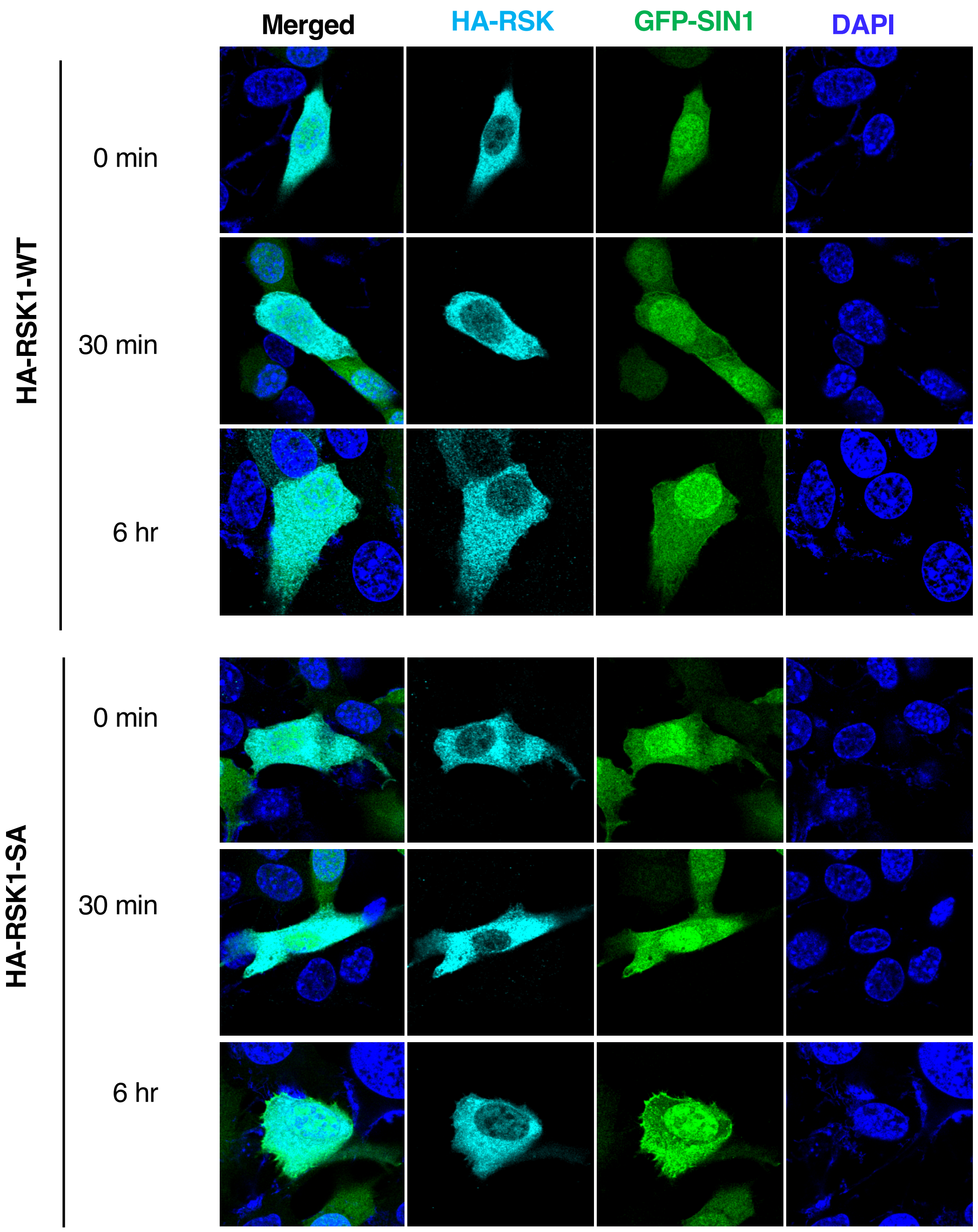

Figure S4: RSK-WT colocalizes with SIN1 at the membrane during early nutrient withdrawal. Growing wild type MEFs were transfected with either HA-RSK1 WT or HA-RSK-Ser381Ala mutant constructs. The following day, cells were resuspended in media lacking or containing glutamine with dialyzed FBS. After the indicated time points of incubation, cells were fixed using 1% PFA, blocked and immunostained for HA to detect HA-RSK-WT or HA-RSK-S381A. DAPI staining indicates the nucleus. Images were acquired on a Leica SP8 confocal laser–scanning microscope and processed using Bitplane Imaris 9.1.2.

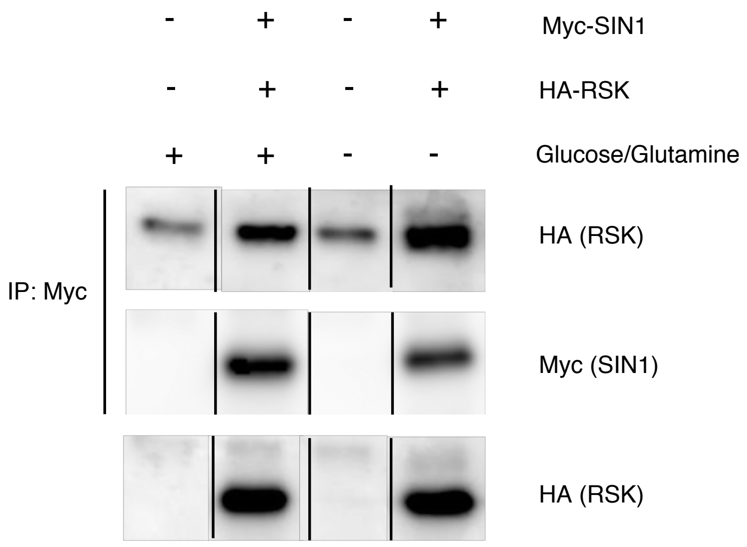

Figure S5: There is increased association of RSK and SIN1 during glucose/glutamine withdrawal. Growing WT MEFs were co-transfected with empty vector or with HA-RSK1 and Myc-SIN1. The Following day, cells were resuspended in DMEM with 10% dialyzed FBS but lacking glucose and glutamine Cells were lysed using CHAPS lysis buffer. Extracts were incubated in Myc antibody overnight. Immunoprecipitates were washed with PBS, fractionated by SDS-PAGE then immunoblotted.for the indicated antibodies. Total HA-RSK levels of extracts (input) were also analyzed.
